# Association of pulmonary lymphocytes with radiation-induced lung disease in a mouse model

**DOI:** 10.1186/s13014-025-02762-0

**Published:** 2025-11-20

**Authors:** Laetitia Sabatier, Aimée-Lee Luco, Mitchell Wiebe, Christina K. Haston

**Affiliations:** 1 Physics Department, I.K. Barber Faculty of Science, The University of British Columbia Okanagan, Kelowna, BC Canada; 2 Meakins-Christie Laboratories, McGill University, Montreal, PQ Canada

**Keywords:** Thoracic irradiation, Pulmonary lymphocytes, Mouse model, Pneumonitis, Fibrosis

## Abstract

**Background:**

Specific congenic mice derived from inbred C3H/HeJ mice, which present early onset pneumonitis, and C57BL/6J mice manifesting later onset pneumonitis with pulmonary fibrosis, vary in time to respiratory distress from these responses following whole thorax irradiation.

**Methods:**

Herein, to investigate a potential adaptive immunity contribution to lung disease in this model, we used flow cytometry to enumerate the pulmonary lymphocytes of C3H/HeJ, C57BL/6J and three lines of sub/congenic mice, at respiratory distress from 18 Gy whole thorax irradiation and in strain matched controls.

**Results:**

CD4 + lymphocyte % of C3H/HeJ mice exceeded that of C57BL/6J mice at both radiation-induced respiratory distress and in unirradiated controls (*P* < 0.04) and pulmonary CD4+% was reduced, at distress relative to control levels, in fibrosis responding strains. CD8 + lymphocyte % was reduced in distressed mice, compared to controls, for 8 of 10 comparisons by strain and sex including those of both pneumonitis and pneumonitis with fibrosis responses. γδ + lymphocyte % was largely unchanged at distress from control levels. Time to radiation-induced respiratory distress, and strain fibrosis score, were each negatively correlated with pulmonary CD4+%, and with the CD4/CD8 ratio, measured in distressed mice (*r*<-0.76; *P* < 0.01) or in unirradiated controls (*r*<-0.75; *P* < 0.02). Inclusion of the lymphocyte profile of unirradiated mice of a separate C3H/HeJ and C57BL6/J-derived congenic line, named *Radpf1* and known to be spared radiation-induced lung disease, yielded a pulmonary CD4+% correlation with time to respiratory distress of (*r* = 0.16, *P* = 0.76) and of (*r*=-0.83; *P* = 0.04) for strain fibrosis score.

**Conclusions:**

Pulmonary lymphocyte profiling revealed strain-dependent %CD4 + lymphocytes, measured in mice manifesting radiation-induced respiratory distress or in untreated controls, to correlate with fibrotic lung disease in this mouse model.

**Supplementary Information:**

The online version contains supplementary material available at 10.1186/s13014-025-02762-0.

## Background

Thoracic radiotherapy can result in the lung tissue injuries of pneumonitis or fibrosis in a susceptible subpopulation of exposed individuals [1]. An improved understanding of the mechanisms driving these responses and of factors affecting susceptibility to these injuries would aid in clinical use of thoracic radiotherapy.

Among the pulmonary responses to radiation, an adaptive immunity contribution to pneumonitis and fibrosis development has been postulated [2, 3] based on established tissue repair responses [4]. In support of this contribution, clinical studies have shown thoracic radiotherapy to increase the lymphocyte % of bronchoalveolar lavage of lung [5] and breast cancer patients [2, 6–8] compared to non-irradiated patients or to healthy controls. Mouse models of radiation-induced lung disease also present increased lavage lymphocyte levels [9–11] and interventions which improved the post irradiation survival of pneumonitis prone C3H/HeJ mice [12] and pneumonitis with fibrosis responding C57BL/6J mice [13, 14] also reduced the numbers of lung tissue lymphocytes. A lymphocyte contribution to radiation-induced lung disease was further shown by Cappuccini et al. [15] who demonstrated Rag2-/- mice, which lack mature T & B cells, to develop enhanced fibrosis compared to genetic background C57BL/6J mice. As T lymphocyte classes, cytotoxic CD8+, helper CD4 + and γδ+, differ in principal immune function [4], the specific lymphocytes triggered by injured tissue have been proposed both to augment or to reduce radiation-induced pneumonitis and fibrosis [3].

In addition to differing in susceptibility to radiation-induced pneumonitis or to pneumonitis with fibrosis, inbred strains of mice vary in time to onset of respiratory distress after whole thorax irradiation [16–18], a trait which has also been assessed clinically [19]. Specifically, our evaluation of the lung injury responses to thoracic irradiation of mice of 27 inbred strains [17] revealed all mice to succumb with pneumonitis, with or without fibrosis, but for the strains to vary in the time post irradiation the respiratory distress from pneumonitis (with or without fibrosis) was evident. We have used the time to onset of respiratory distress, or survival time, as a phenotype of radiation-induced lung disease. C3H/HeJ and C57BL/6J mice differ in time to respiratory distress after whole thorax irradiation [10, 17, 18, 20, 21] which is 10–14 weeks for C3H/HeJ mice and 22–30 weeks for C57BL/6J mice and genetic mapping in offspring of these strains was used to identify a locus on chromosome 2 affecting survival time post thoracic irradiation [10]. In that study [10], we showed the post-irradiation survival time of mice to negatively correlate with extent of pneumonitis, but not of fibrosis. The effect of the chromosome 2 locus on time to respiratory distress was subsequently verified with breeding and phenotyping the thoracic radiation response of congenic [22] and subcongenic [23] mice. In the latter study [23], we identified mice of a panel of chromosome 2 subcongenics to vary in time to respiratory distress from radiation-induced pneumonitis and fibrosis and for chromosome 17 subcongenic mice, named *Radpf1* for radiation-induced pulmonary fibrosis-1, to survive to the experimental endpoint, with limited pneumonitis and fibrosis. Bioinformatic analyses of the chromosome 2 & 17 candidate genes suggested an adaptive immunity contribution to the varying radiation induced lung disease susceptibility of the mice.

In this study, we investigated the hypothesis that pulmonary lymphocyte profiles are associated with the onset of distress from radiation induced lung disease, and the extent of fibrosis, in this mouse model. To test this hypothesis, we enumerated pulmonary lymphocytes in groups of male and female mice of this 5-strain panel at each of (i) baseline (pre-irradiation), (ii) experimental weeks 14–22 of unirradiated mice and (iii) the onset of strain-dependent respiratory distress from thoracic irradiation, and assessed the lymphocyte association to the reported radiation-induced lung phenotypes [22, 23].

## Methods

### Mice

C57BL/6J and C3H/HeJ mice were purchased from the Jackson Laboratory (Bar Harbor, ME) and housed in the animal facilities of the Meakins-Christie Laboratories and of the McGill University Health Centre. One line of congenic and two lines of subcongenic mice with specific C3H/HeJ donor regions in a C57BL/6J genetic background (depicted in Additional File 1), described in [23], were bred and housed at the McGill University Health Centre. The whole thorax irradiation-induced lung phenotypes of these lines of mice, from [23], are listed in Additional File 1. As indicated, the congenic and subcongenic mice were previously shown to have developed pneumonitis and fibrosis at respiratory distress. Mice of the chromosome 17 congenic line, (*Radpf1*, ref 23), were also bred and housed at the McGill University Health Centre. All mice were handled according to protocol 5610 approved by the Animal Care Committee at McGill University, in accordance with regulations set by the Canadian Council on Animal Use and Care.

### Radiation exposure and experimental time points

At 8–10 weeks of age, mice were exposed to a single dose of whole-thorax irradiation (18 Gy; dose rate 0.54 Gy/minute) using a Faxitron CP-160 X-ray machine, as previously described [12, 22–26]. Beginning eight weeks after irradiation, mice were weighed weekly to monitor the onset of respiratory distress. Mice were euthanized when in respiratory distress or at the end of the experiment, which was 26 weeks post-irradiation for female mice and 35 weeks post-irradiation for male mice. Mice were considered to be in distress when they exhibited symptoms of greater than 20% loss in weight, hunched posture, rapid breathing, lethargy, and ruffled fur. Mice were assessed for survival post irradiation and flow cytometric analysis of pulmonary lymphocyte populations was completed. Groups of unirradiated mice of each strain were euthanized at baseline (8 weeks of age) or at the 14–15 week or 22 week timepoint for pulmonary flow cytometry. Unirradiated female *Radpf1* mice were euthanized at 14 weeks of age.

### Lymphocyte profiling

At sacrifice, lung tissue was collected, cut into small pieces and digested at 37 °C for 45 min with 1 mg/ml of Collagenase D and 0.1 mg/ml of DNase (Roche, Mississauga, ON). Following digestion, a single cell suspension was obtained by mechanical disruption of the tissue using a cell dissociation sieve and tissue grinder (Sigma-Aldrich, Oakville, ON) and a 70 μm cell strainer. Red blood cells were lysed with red blood cell lysis buffer (Biolegend, San Diego, CA). Total cells were counted on a Coulter Ac·T diff Analyser (Beckman Coulter, Mississauga, ON). Cells were stained with fixable viability dye efluor 780 (eBioscience). The cells were washed and stained with surface antibodies against CD3 (eBioscience, San Diego, CA), CD4 (eBioscience), CD8 (eBioscience), and TCRγδ (BD Biosciences). Cells were acquired using a BD LSRII cytometer and analysis was conducted using FACS Diva and FlowJo software (Ashland, OR). The employed gating strategy is depicted in Additional File 2.

### Statistical analysis

Differences in post-irradiation survival time were analyzed by log rank test. Differences in cell type % between irradiated mice and control mice were assessed by t test. The phenotypes of average time from irradiation to the onset of respiratory distress or “survival time”; and fibrosis score, defined as the % of lung tissue with a fibrotic scar, for each mouse line were taken from previous work characterizing the radiation response of these lines [23]. Phenotypes for male C57BL/6J and male C3H/HeJ mice were taken from [22]. Correlations between cell type % and survival or fibrosis phenotypes were assessed in Excel and lines of best fit, with 95% confidence intervals, produced using the regplot function within the seaborn module of Python.

## Results

To investigate whether distinct adaptive immune profiles associated with the varied time to onset of radiation-induced pulmonary distress, a panel of congenic, subcongenic, and mice of parental C3H/HeJ and C57BL/6J strains, was treated with whole-thorax irradiation and monitored for response.

**Fig. 1:**
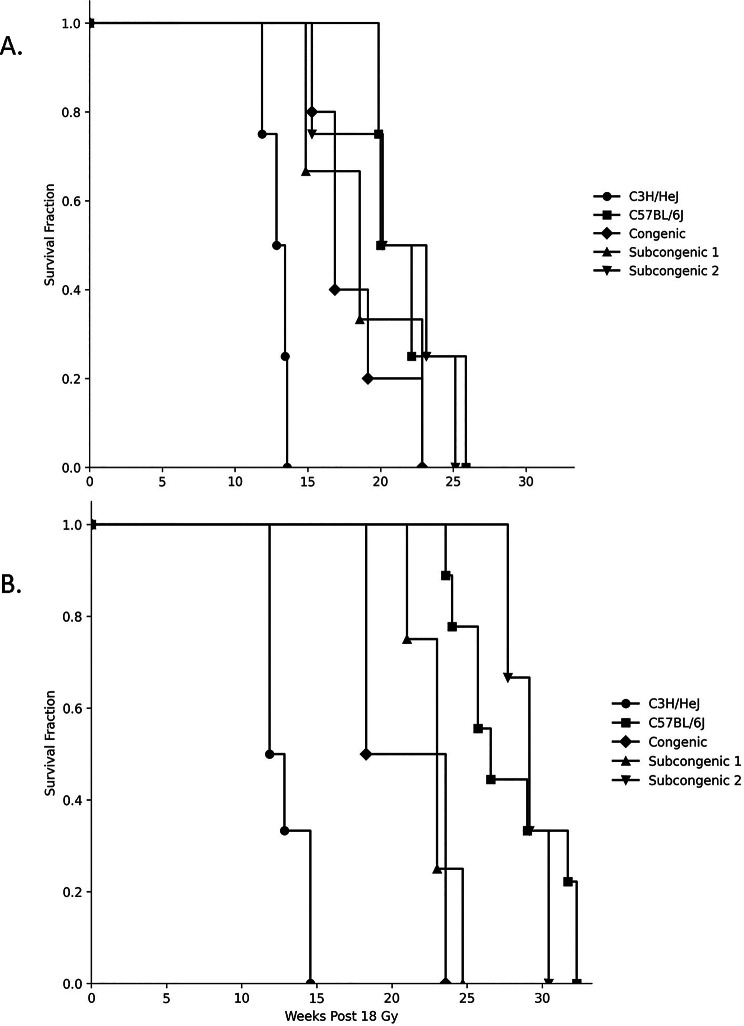
Survival from respiratory distress of C57BL/6J, C3H/HeJ, congenic and subcongenic mice exposed to thoracic radiation. Mice were exposed to 18 Gy whole-thorax irradiation and euthanized when in respiratory distress or at the end of the experiment, which was 26 weeks post-irradiation for female mice and 35 weeks post-irradiation for male mice. Survival of **A**. female and **B**. male mice; *n* = 3–9 mice per group

As before [17, 18, 22], female C57BL/6J mice manifested distress from whole thorax irradiation at an earlier time, and thus had a shorter survival time, than males of the same strain (*P* = 0.005). The data of Fig. 1 are, therefore, shown separately for each sex. Also consistent with prior reports [10, 17, 18, 20, 21], the survival time of C3H/HeJ mice in response to whole thorax irradiation was reduced compared to that of C57BL/6J mice (*P* < 0.007). As shown in Fig. 1A, the times to the onset of distress, or survival time, of female mice of each of the five strains agreed (*P* >0.26) with those identified in our prior study (ref [23], reproduced in Additional File 1). For males, as shown in Fig. 1B, the post-thoracic irradiation survival times of C3H/HeJ and C57BL/6J mice agreed (*P* >0.27) with those of a previous report [22] wherein mice were treated with the same radiation protocol. For males of the congenic and subcongenic lines, respiratory distress occurred earlier in mice of the present study, exposed to 18 Gy by a 160 kVp Faxitron instrument, than in the previous work completed with a treatment of 16 Gy by a 6MV linear accelerator [23], resulting in survival differences between studies for male congenic mice (*P* = 0.019), and subcongenic line 2 mice (*P* = 0.055) while for subcongenic line 1 mice the survival comparison yielded (*P* = 0.33). Overall, the strain order for onset of distress following thoracic irradiation of C3H/HeJ < congenic = subcongenic line 1 < subcongenic line 2 = C57BL/6J, for each of male and female mice agrees with the prior report (ref [23]; Additional File 1).

**Fig. 2:**
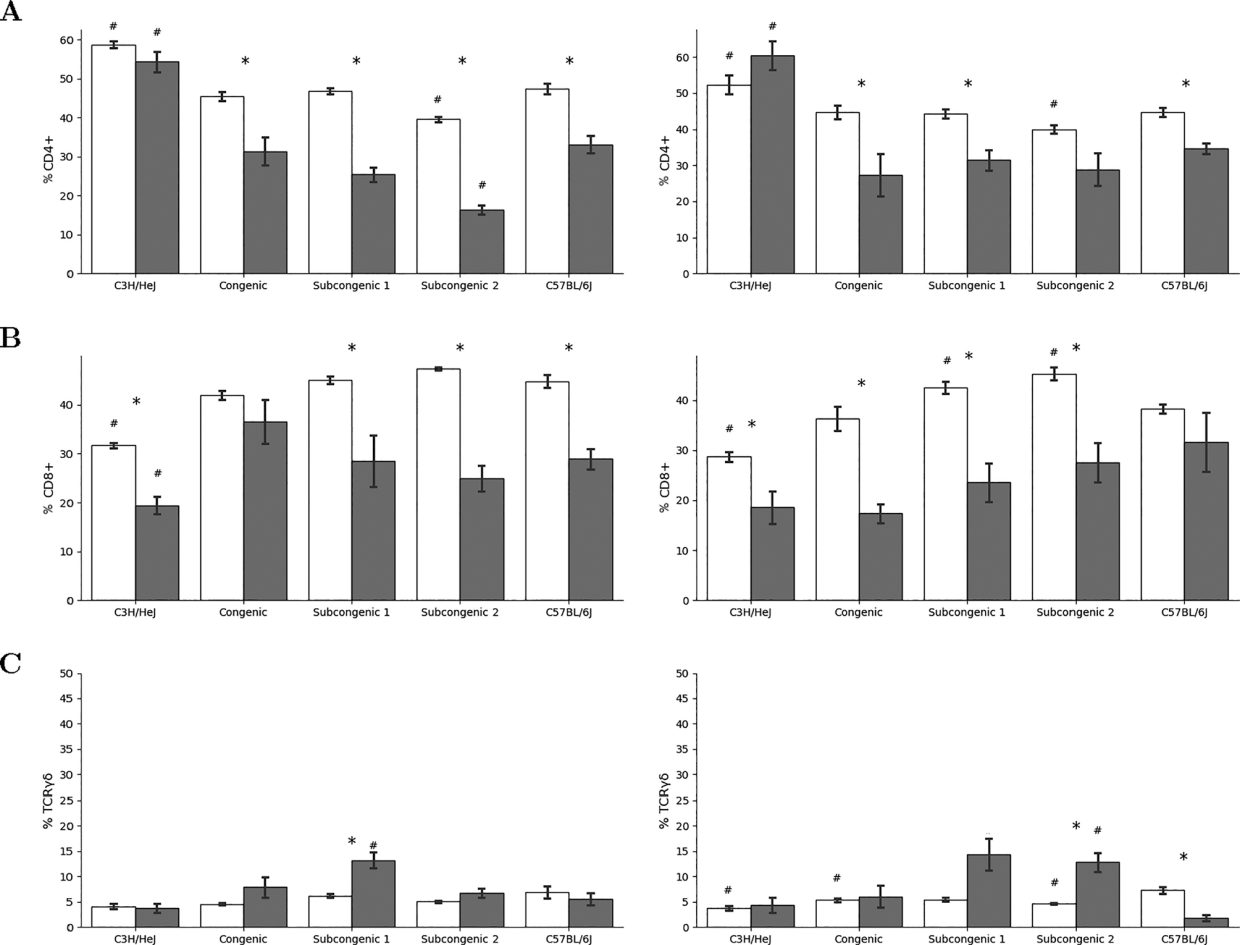
Pulmonary CD4+, CD8 + γδ + lymphocyte populations of mice at radiation-induced respiratory distress. Mice were exposed to 18 Gy thoracic irradiation and euthanized when presenting respiratory distress (grey bars). Control mice were not irradiated and were euthanized at experimental week 14–15 or 22 (open bars). Cell counts were measured through flow cytometry of total lung tissue. Percentage of (**A**) CD4+, (**B**) CD8 + and (**C**) γδ + cells in CD3 + population in the lung for male (left) and female (right) mice. Results are mean ± SEM for groups of 3–10 mice. * indicates a significant difference between irradiated and control mice of the same strain, *P* < 0.05. # indicates significantly different from C57BL/6J mice, *P* < 0.05

We enumerated the pulmonary CD4+, CD8 + and γδ + lymphocyte populations of mice in respiratory distress and of untreated control mice. Consistent with previous reports [18, 27], the %CD4 + pulmonary lymphocytes of untreated C3H/HeJ mice exceeded that of C57BL/6J mice, as shown in Fig. 2A, and this strain difference was also apparent in mice in distress from whole thorax irradiation. Further, CD4 + lymphocyte percentages were significantly decreased in the lungs of distressed mice, compared to levels in controls, in all fibrosis responding strains (Fig. 2A), with the exception of subcongenic line 2 females (*p* = 0.09) while CD4 + lymphocyte percentages were not changed in the lungs of distressed C3H mice, compared to controls, (*p* >0.14).

In the lungs of unirradiated mice, CD8+% of C57BL/6J mice exceeded that of C3H/HeJ mice (Fig. 2B) and this strain difference was evident in male mice at distress (*P* = 0.004) and not in females (*P* = 0.12). The percentages of CD8 + lymphocytes significantly decreased in the lungs of distressed mice, compared to controls, for 8 of the 10 strain and sex groupings evaluated (Fig. 2B) which included lines of mice both developing, and not developing, fibrosis. There was also a greater γδ + lymphocyte% in unirradiated female C57BL/6J mice than in female C3H/HeJ mice (Fig. 2C) and for male mice the strain comparison yielded *P* = 0.06. The γδ + lymphocyte% did not differ (*P* > 0.26) between C3H/HeJ and C57BL/6J mice in respiratory distress from whole thorax irradiation. Percentages of γδ + lymphocytes in the lungs of distressed mice, were unaltered compared to controls, for 7 of the 10 strain and sex groupings evaluated (Fig. 2C). There were no radiation-induced changes in pulmonary CD4+, CD8 + and γδ + lymphocyte percentages, compared to untreated controls, which were common strains developing the early onset distress response (C3H/HeJ, congenic & subcongenic line 1) and distinct from those developing the later onset distress response (C57BL/6J and subcongenic line 2).

**Fig. 3:**
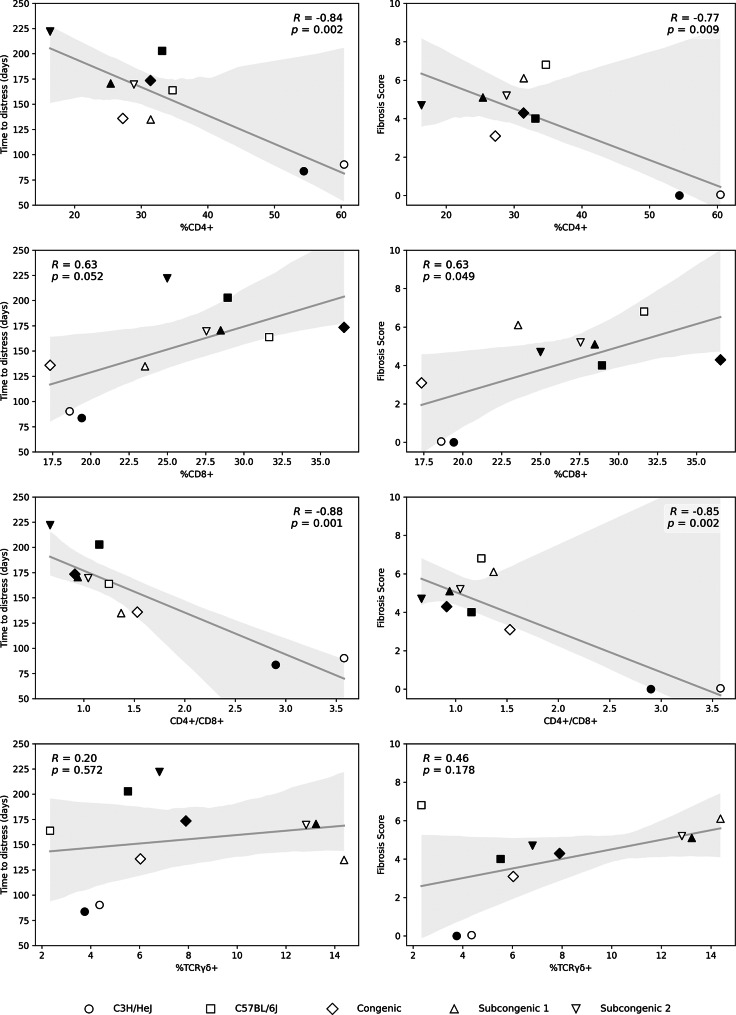
Post irradiation survival time and fibrosis response are correlated with pulmonary lymphocyte percentages in distressed mice. The average lymphocyte % in irradiated mice was taken from Fig. 2. The average survival time and fibrosis score for groups of mice by strain and sex were taken from [22, 23] and are listed in Additional File 1. Data of male mice indicated by filled symbols, data of female mice by open symbols. Line of best fit, shaded 95% confidence region

To determine whether any pulmonary lymphocyte percentage, measured at radiation-induced respiratory distress, is a potential component of the pathology in this model, correlations of lymphocyte % with the phenotypes of survival time and fibrosis score were evaluated using means for each trait, by strain and sex. As shown in Fig. 3, each of the onset of respiratory distress (survival time) and fibrosis score was significantly negatively correlated with %CD4 + lymphocytes and with the CD4+/CD8 + ratio in the lung.

**Fig. 4:**
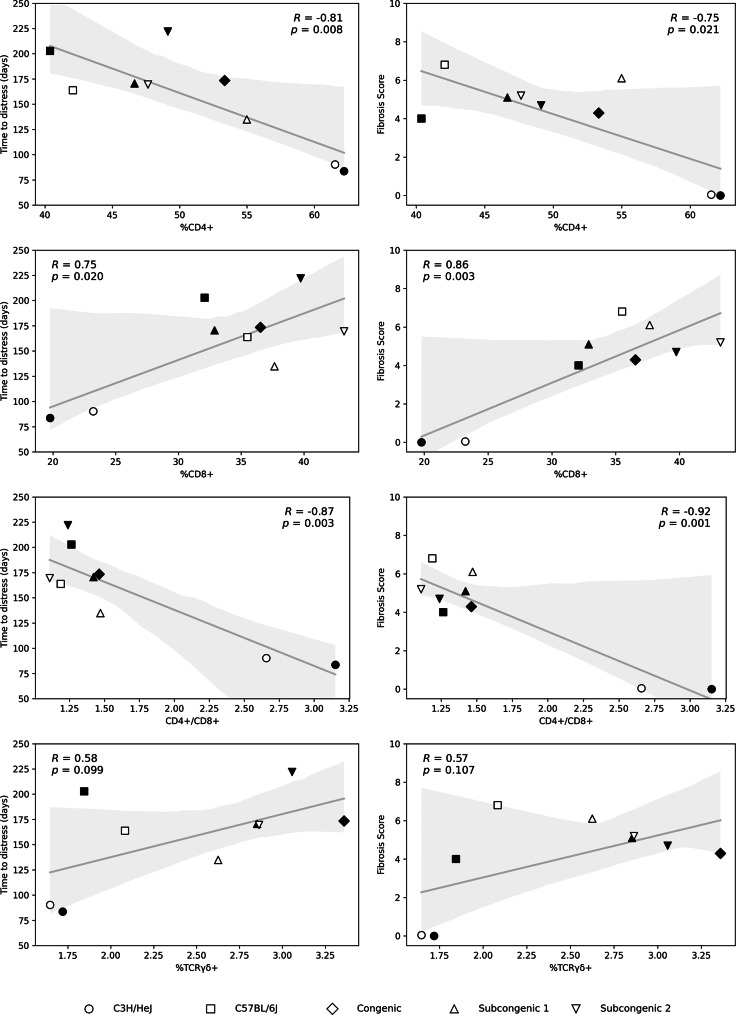
Post irradiation survival time and fibrosis response are correlated with pulmonary lymphocyte percentages of unirradiated mice at baseline. Mice (*n* = 5–6 per group) were not irradiated and were euthanized at 8 weeks of age. Cell counts were measured through flow cytometry of total lung tissue. The average survival time and fibrosis score for groups of mice by strain and sex were taken from [22, 23] and are listed in Additional File 1. Data of male mice indicated by filled symbols, data of female mice by open symbols. Line of best fit, shaded 95% confidence region

Next, to investigate whether lymphocyte percentages were apparent in untreated mice which indicated later known strain responses to thoracic irradiation, in terms of survival time or of % fibrosis, correlations of lymphocyte percentages in baseline (8 week old, pre irradiation) mice to average strain phenotypes post irradiation were evaluated. As shown in Fig. 4, %CD4+, %CD8 + and the ratio of CD4+/CD8 + lymphocytes in lungs of untreated mice, at baseline, were each significantly correlated with post thoracic irradiation survival time. Secondly, known fibrosis score increased with increasing baseline CD8 + lymphocyte percentage, and with decreasing %CD4+, & CD4+/CD8 + lymphocyte ratio, measured in the lungs of unirradiated mice at baseline. The significant associations of lymphocyte percentages to survival time and to fibrosis score presented in Fig. 4 were also evident using the data of the untreated controls (age 23–30 w) from Fig. 2, as shown in Additional File 3.

Finally, we enumerated the pulmonary lymphocytes of six unirradiated female *Radpf1* mice, a separate C3H/HeJ and C57BL6/J derived congenic line, for which we previously identified 19/20 mice treated with 16 Gy whole thorax irradiation to be spared respiratory distress at 25 weeks after treatment and to manifest a reduced fibrosis score of 0.8% of the lung at the experimental endpoint [23]. To determine whether the lymphocyte profile of *Radpf1* mice was consistent with that of genetic background C57BL/6J mice, or influenced by the C3H/HeJ alleles of the donor region, strain comparisons were completed. The %CD4+ (54.6 ± 0.3) and %γδ+ (4.2 ± 0.5) lymphocytes in lungs of *Radpf1* mice did not differ from levels in female C3H/HeJ controls of Fig. 2 (*P* >0.42) indicating C3H/HeJ alleles in the donor region may affect these lymphocyte traits, and %CD8 (33.6 ± 0.7) exceeded that of C3H/HeJ females (*P* = 0.003). The lymphocyte profile of *Radpf1* mice differed from the profile of female C57BL/6J controls shown in Fig. 2 (*P* < 0.007).

**Fig. 5:**
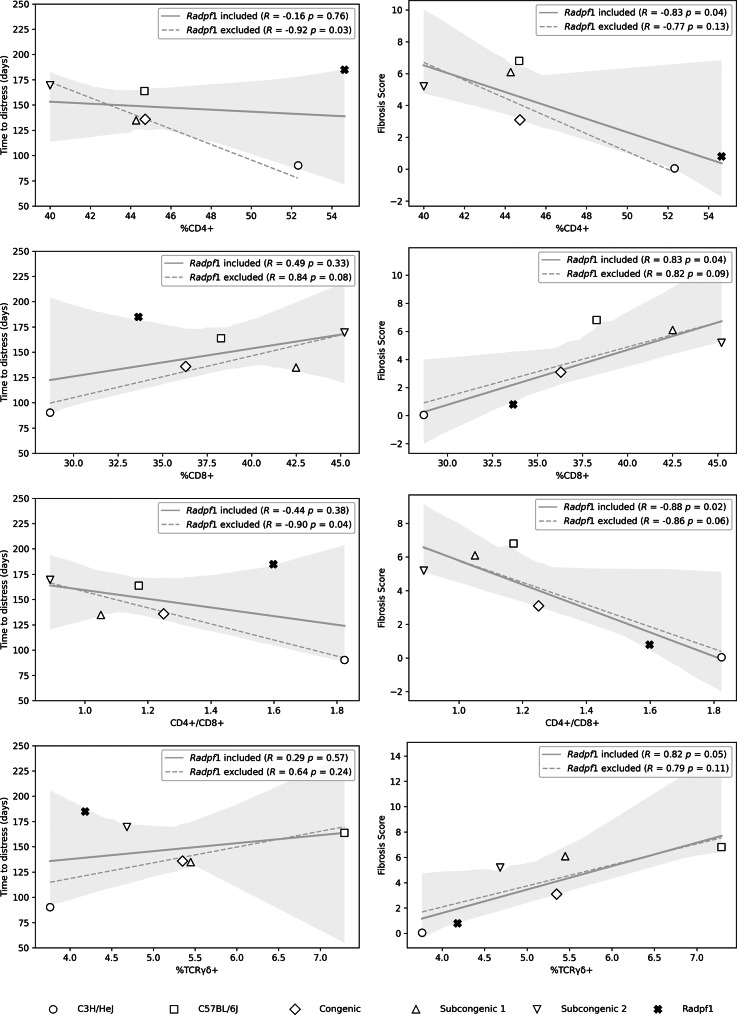
Lymphocyte traits of *Radpf1* mice alter correlations to post irradiation survival time and fibrosis response. Female *Radpf1* mice (*n* = 6) were not irradiated and were euthanized at 14 weeks of age. Cell counts were measured through flow cytometry of total lung tissue. Data for *Radpf1* mice are represented with an **x**. The average lymphocyte % in remaining untreated control mice taken from Fig. 2. The average survival time and fibrosis score for groups of female mice by strain were taken from [23] and are listed in Additional File 1. Line of best fit, shaded 95% confidence region

An assessment of pulmonary lymphocyte association with known strain response of survival time post thoracic irradiation, completed with data from control female mice only, revealed the correlations of %CD4+, %CD8 + and the ratio of CD4+/CD8 + to change from significance of *P* < 0.08 to *P* > 0.32 with the inclusion of data from *Radpf1* mice, as shown in Fig. 5. Secondly, the lymphocyte correlations to known fibrosis score improved from *P* < 0.13 to *P* < 0.05 with the inclusion of data from *Radpf1* mice.

## Discussion

In this work we demonstrate the different radiation-induced lung responses, of early onset pneumonitis in C3H/HeJ mice, and later onset pneumonitis with pulmonary fibrosis of C57BL/6J mice, to correlate with the CD4/CD8 pulmonary lymphocyte profile of the strains. The strain-dependence in pulmonary CD4% was revealed to associate with extent of radiation-induced pulmonary fibrosis, and, we identified the lymphocyte profile in the lungs of untreated mice to correlate with the known strain responses to thoracic irradiation.

The association of pulmonary lymphocytes with radiation-induced lung disease in this model agrees well with prior experimental work [15, 28] and with clinical reports [29, 30]. Indeed, the decrease in pulmonary CD4% in mice in respiratory distress with radiation-induced fibrosis extends findings of others wherein CD4% was experimentally reduced in C57BL/6J mice resulting in increased fibrosis, following thoracic irradiation [15]. Paun et al. [18], using the C57BL/6J model of radiation-induced lung disease, further showed that within CD4 + lymphocytes, T helper type 17 cells augmented and T helper type 1 cells spared, the fibrosis response. The identified associations of increasing pulmonary CD8 + lymphocytes, and decreasing CD4/CD8 ratio, each with increasing fibrosis score in mice, are supported by similar correlations reported with lavage lymphocytes of patients with idiopathic pulmonary fibrosis [29]. Additional evidence of an increase in pulmonary levels of CD8 + cells with increased fibrosis is provided by Feng et al. [28] who showed the adoptive transfer of CD8 cells from mice with bleomycin-induced pulmonary fibrosis to augment pulmonary fibrosis in recipient mice. Clinically, the CD4/CD8 ratio may be used to indicate infection (if elevated) or compromised immunity (if inverted) although the normal range of 1.5–2.5 is described as poorly defined [31]. In the present work, an increased pulmonary CD4/CD8 ratio was associated with earlier onset of respiratory distress from thoracic radiation exposure in mice, as has been reported in the clinical lung response of sarcoidosis. In detail, Kullberg et al. [30], who describe sarcoidosis to commonly include a T cell alveolitis, and an elevated bronchoalveolar lavage CD4/CD8 ratio, report a decreased CD4/CD8 ratio as an indicator of patient response to therapy, in line with the pneumonitis response reported here. In patients exposed to thoracic cavity radiotherapy, however, both increased [8] and decreased [7] bronchoalveolar lavage CD4/CD8 ratios, compared to those of untreated controls, have been reported, thus a link of these measures to lung disease outcome, if any, remains undefined.

Inherent differences in lymphocyte levels may have utility as a predictive assay of radiation-induced lung disease, as has been observed in a limited clinical study [32]. Specifically in the current work, in addition to pulmonary lymphocyte measures completed in mice in respiratory distress, we evaluated mice at baseline and untreated controls at experimental timepoints and revealed lymphocyte associations to previously measured radiation injury responses. Further, untreated *Radpf1* mice, which are spared radiation-induced lung fibrosis and which have C3H/HeJ alleles on chromosome 17, were shown to have pulmonary CD4 lymphocyte % as in C3H/HeJ mice, supporting the pre-treatment association of pulmonary CD4 with the trait of radiation induced pulmonary fibrosis in this mouse model. These data suggest that chromosome 17 donor region C3H/HeJ alleles influence the C3H/HeJ: C57BL/6J strain dependence in pulmonary CD4 lymphocytes [18, 27]. Supporting this possibility, He et al. [33], identified levels of circulating CD4 cells to be influenced by genetic variation in this donor region in collaborative cross mice.

As lymphocytes have been shown to affect the macrophage phenotype in related lung infection models [34], strain dependent CD4 + cell numbers may have altered radiation-induced lung pathology by affecting the macrophage response. Supporting this possibility, the radiation-induced lung disease of C3H/HeJ and C57BL/6J mice has been shown to differ in caspase-3 positive [35] and mac-3 positive [36] macrophage infiltration. Further investigation by Groves et al. [37], documented C3H/HeJ: C57BL/6J strain differences in alveolar and infiltrating macrophage numbers in the lungs of mice exposed to thoracic irradiation, however, a lymphocyte influence on the macrophage phenotype in radiation-induced lung disease was not investigated in these studies or in the present work. A sex difference in survival time was evident as female mice manifested respiratory distress earlier, post thoracic irradiation, than did males, as in prior work [10, 22]. An influence from sex has been reported in other radiation responses [38], including survival post total body irradiation where female non human primates have higher mortality, than males, at the same radiation dose [39]. The reason for the differential onset in radiation response is not known but sex differences in body size and hormone levels have been suggested to affect these traits [38].

Herein, lymphocyte profiles were procured from lung tissue of untreated mice or after mice received whole thorax irradiation and include certain limitations. The profiled tissue included circulating blood and therefore strain or radiation treatment dependent differences in pulmonary blood levels, if occurring, would have affected reported lymphocyte measures. In a comparison of lung processing techniques, however, Singer et al. [40] found no difference in numbers of haemopoietic cells assayed by flow cytometry between lungs of mice processed with saline flushing and those without flushing. Secondly, whole lung irradiation of mice was used as a model of the pathological response of the lung to radiation treatment, and although, in mice, the response of the whole lung is retained in exposures of one lung [41] or of an upper lobe only [42], the model may not reflect the clinical condition of irradiating restricted lung volumes.

## Conclusions

In summary, through correlative analysis we demonstrate an association between pulmonary lymphocyte profiles from untreated control mice, or from mice in respiratory distress from whole thorax irradiation, and resultant disparate lung disease in a C3H/HeJ and C57BL/6J mouse model. Congenic region influence on inbred strain variation in pulmonary CD4% was revealed which, as it may affect radiation induced lung disease, is a potential pre treatment biomarker of radiation response.

## Supplementary Information

Below is the link to the electronic supplementary material.

Supplementary Material 1

## Data Availability

The dataset(s) supporting the conclusions of this article is(are) included within the article (and its additional file(s)).
