# Supplementary material for: Association of pulmonary lymphocytes with radiation-induced lung disease in a mouse model
Source: Radiat Oncol. 2025 Nov 20;20:172. doi: 10.1186/s13014-025-02762-0 (PMC12632038; doi:10.1186/s13014-025-02762-0)
Supplement: Supplementary file 1 — Supplementary Material 1 [file 13014_2025_2762_MOESM1_ESM.pdf]

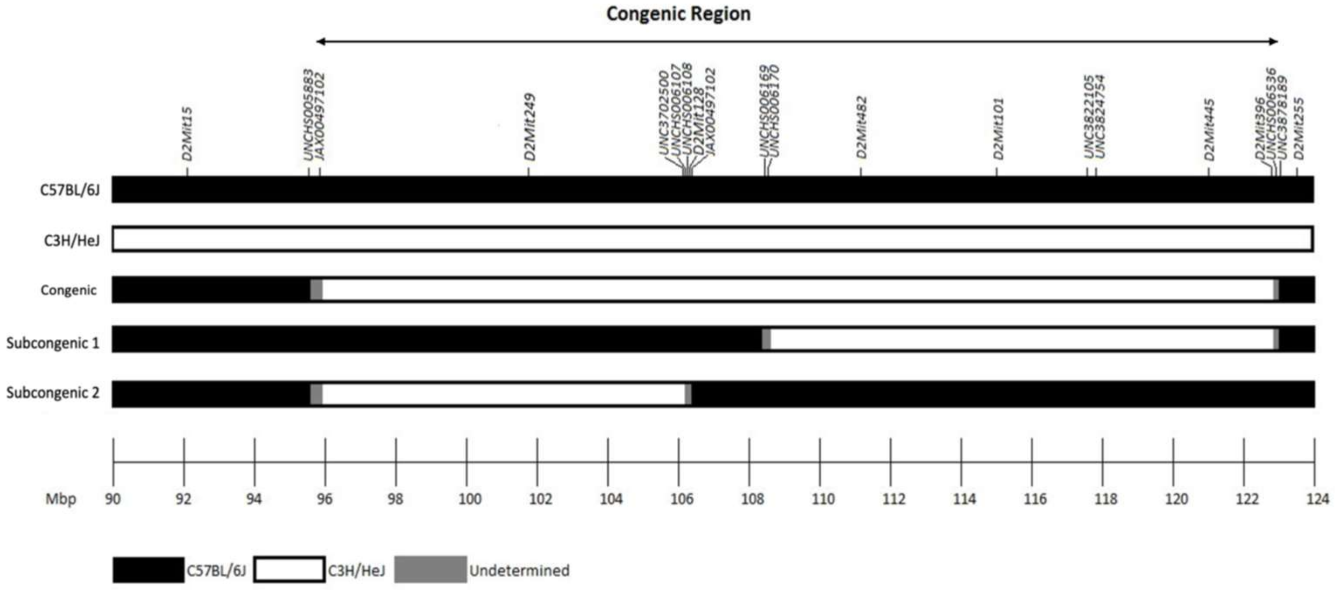

Strain	Sex	Survival Time (days)	Fibrosis Score (%)	Pneumonitis Score
C57BL/6J	male	202.8 ± 11.1	4.0 ± 1.7	4.4 ± 0.4
	female	163.8$^{\#}$ ± 4.3	6.8 ± 1.0	4.3 ± 0.2
C3H/HeJ	male	83.8* ± 3.9	0* ± 0	4.4 ± 0.2
	female	90.2* ± 1.2	0.05*$^{\#}$ ± 0.02	3.2*$^{\#}$ ± 0.1
Congenic	male	173.5* ± 7.2	4.3 ± 1.1	3.9 ± 0.3
	female	136.0*$^{\#}$ ± 5.0	3.1* ± 0.5	4 ± 0
Subcongenic 1	male	170.6* ± 5.7	5.1 ± 1.5	4.5 ± 0.2
	female	134.9*$^{\#}$± 3.6	6.1 ± 1.6	4.1 ± 0.2
Subcongenic 2	male	222.1 ± 7.1	4.7 ± 1.8	3.3 ± 0.4
	female	169.5$^{\#}$ ± 1.6	5.2 ± 1.1	3.2* ± 0.1

data are mean ± standard error of the mean
*different from sex matched C57BL/6J mice (P<0.05)
$^{\#}$different, by sex, within strain (P<0.05)

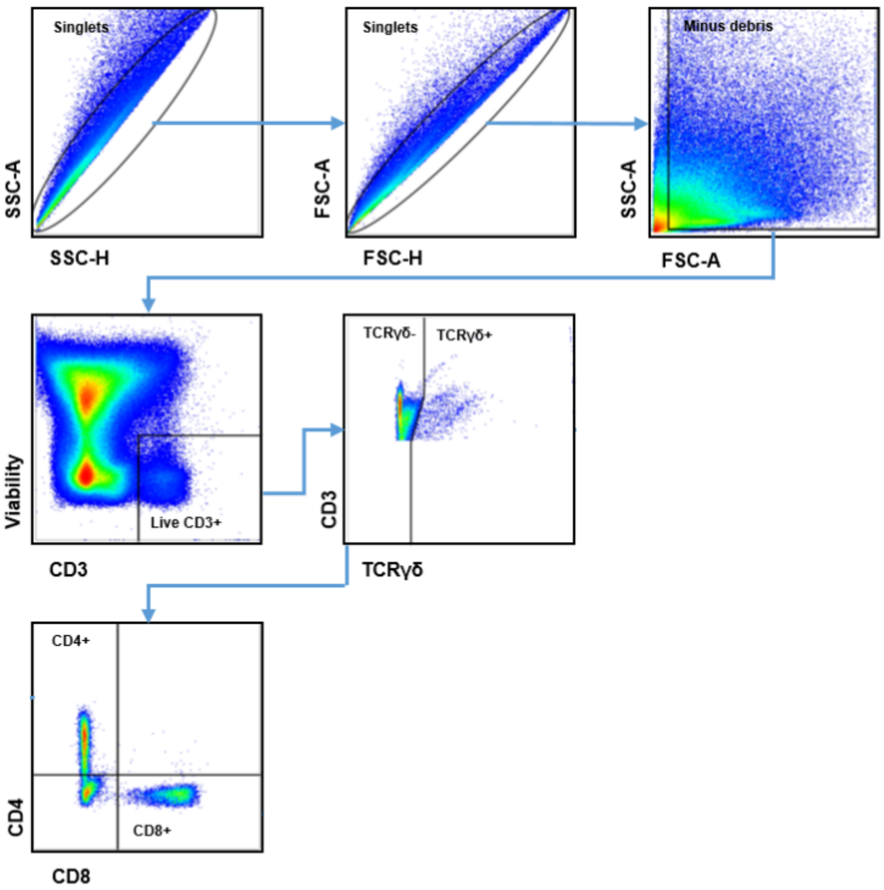
Singlets
SSC-A
SSC-H
Singlets
FSC-A
FSC-H
Minus debris
SSC-A
FSC-A
Viability
Live CD3+
CD3
TCRγδ-
TCRγδ+
CD3
TCRγδ
CD4+
CD8+
CD4
CD8

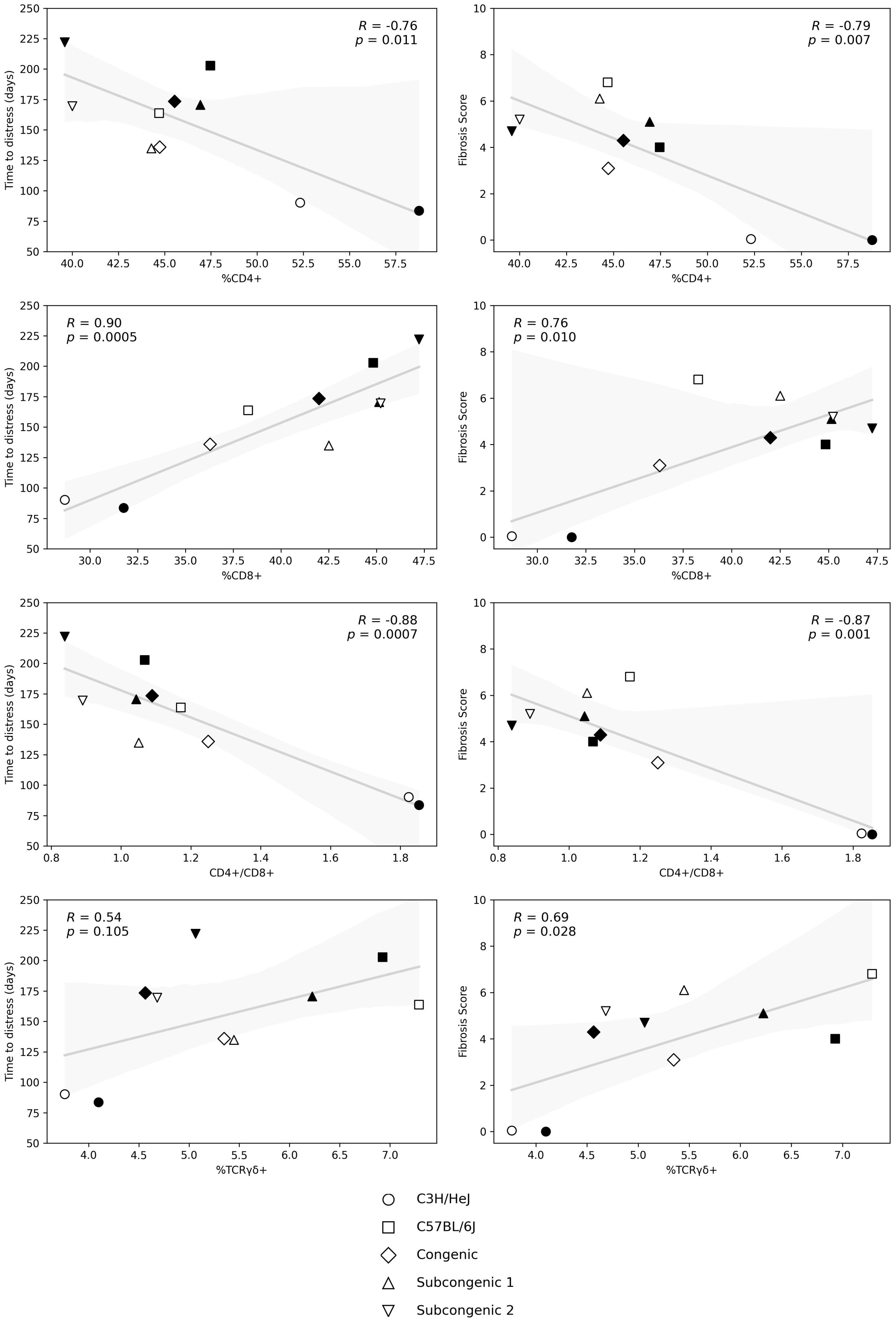
R = -0.76
p = 0.011
R = -0.79
p = 0.007
R = 0.90
p = 0.0005
R = 0.76
p = 0.010
R = -0.88
p = 0.0007
R = -0.87
p = 0.001
R = 0.54
p = 0.105
R = 0.69
p = 0.028
Time to distress (days)
Fibrosis Score
%CD4+
%CD8+
CD4+/CD8+
%TCRγδ+
C3H/HeJ
C57BL/6J
Congenic
Subcongenic 1
Subcongenic 2
